# Non-surgical Removal of Dens Invaginatus in Maxillary Lateral Incisor Using CBCT: Two-year Follow-up Case Report

**DOI:** 10.1515/med-2019-0089

**Published:** 2019-10-19

**Authors:** Babita Pradhan, Yuan Gao, Libang He, Jiyao Li

**Affiliations:** 1 State Key Laboratory of Oral Diseases & National Clinical Research Center for Oral Diseases & Department of Cariology and Endodontics West China Hospital of Stomatology, Sichuan University, #14, 3rd Section of RenMin South Road,Chengdu 610041, Chengdu, China

**Keywords:** Dens invaginatus, Dental operating microscope, Ultrasonics, Cone-beam computed tomographic imaging, Dens invaginatus removal

## Abstract

A 14-year female presented with an atypical looking tooth #7 with a sinus tract on tooth #8. A gutta-percha point inserted into the sinus tract confirmed the affected tooth #7. A radiographic examination of tooth showed a lateral radiolucency with respect to tooth #7. Cone-beam computed tomographic imaging was done for the three-dimensional reconstruction analysis. Dens invaginatus (Oehler’s type III) with pulp necrosis and chronic apical periodontitis was the definitive diagnosis. Use of the dental operating microscope and ultrasonics helped in the removal of the invaginated structure. At the two year follow-up, no clinical and radiographic evidence of infection was observed.

## 1 Introduction

Dens invaginatus (DI) is a developmental aberration of the teeth with an early invagination of the enamel and dentine extending deep into the pulp cavity and to the roots [1]. DI may occur in any deciduous or permanent tooth, but the most commonly affected teeth are the maxillary central and lateral incisors [2]. The most popular classification of DI is described by Oehlers [3] which is based on the extent of apical migration of the invagination.

Type I – The invaginatus confines within the crown not extending beyond the cementoenamel junction.

Type II –The invaginatus invades the root but remains confined as a blind sac which may or may not have communication with the dental pulp.

Type III – The invaginatus invades the root perforating the apical area exhibiting an extra foramen in the apical or periodontal area. Also, the invaginatus has no immediate communication with the pulp.

The clinical significance of DI is the increased risk of oral microbial contamination through the coronal aspect of the invagination, leading to infection of the root canal system [4]. Various clinical approaches including restorative procedures, nonsurgical endodontic treatment, endodontic surgery, intentional replantation or even teeth extraction have been described to treat DI [5, 6, 7, 8]. However, there are many studies which have reported the removal of the DI [9-14].

Furthermore, to appreciate the complexity of the anomaly, radiographs at different angulations are essential. Cone-beam computed tomographic imaging (CBCT) can also be useful for assessing these teeth [15]. Use of the CBCT and dental operating microscope (DOM) in clinical endodontic practice has increased the feasibility and knowledge regarding various anatomical complexities. This case presents an overview of the practicability of using CBCT in the diagnosis and treatment planning, along with DOM and ultrasonics in the nonsurgical endodontic management of the DI.

## 2 Case report

**Figure 1:**
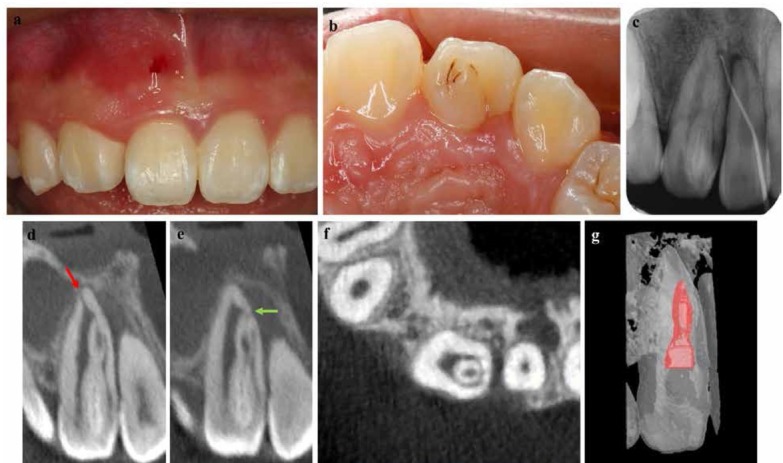
(a) Sinus tract with respect to tooth #8. (b) Unusual prominent lingual cusp and a deep lingual pit with respect tooth #7. (c) IOPA showing the sinus tract done by GP cone confirming the lesion on tooth#7. (d) CBCT image of the invaginated structure showing the main apical foramen (red arrow). (e)Axial view of CBCT image showing the invaginated with separate portal exit(green exit). (f) Axial view of CBCT showing the invaginated structure. (g) CBCT 3D reconstruction images demonstrating the invaginated structure.

A 14-year-old female was referred for evaluation and treatment to the department of endodontics. She complained of painful swelling in the right maxillary region for two months. Her medical history was noncontributory. Clinical examination revealed a sinus tract associated with tooth #8 (Fig.1a), while the clinical crown of tooth #7 was unusual with a pronounced lingual cusp and deep lingual pit (Fig. 1b). Tooth #7 was tender to vertical percussion with normal mobility scored as 1° mobility (less than 1mm) and periodontal probing depths less than 3 mm. Tooth #7 did not respond to a cold thermal test (Endo Ice, Coltene Whaldent Inc, Cuyahoga Falls, OH) and electric pulp tester (Denjoy, China). However, tooth #8 was responsive to cold thermal test and electric pulp tester and not tender to percussion or palpation indicating normal pulp. The ISO #40.02 gutta-percha (GP, Dentsply, Maillefer) was inserted to full depth through the sinus opening and an intraoral periapical radiograph (IOPA) was taken. The GP followed the lesion tract and ended at the apex of tooth #7 which confirmed the lesioned tooth (Fig. 1c)

A radiographic examination of tooth #7 showed a lateral radiolucency and revealed an anomalous internal structure consistent with a DI. A CBCT scan with a CBCT system (3D Accuitomo; Morita, Kyoto, Japan) was done to better visualize the tooth structure with DI in three dimensions. The CBCT images demonstrated the DI was entirely enclosed in the pulp canal space and had a separate portal exit (Fig.1d). It also revealed that the body of DI was almost a separate entity from the main root canal (Fig.1e and 1f). MeVisLab(MeVis Research, Bremen, Germany) was used for the three-dimensional (3D) reconstruction and analysis of the CBCT images (Fig.1g).

Tooth #7 was diagnosed as DI (Oehler’s type III) with pulp necrosis and chronic apical periodontitis. Endodontic treatment and microscopic removal of DI was planned, and the possibility of the endodontic surgical intervention was also explained to the patient’s parents.

**Figure 2:**
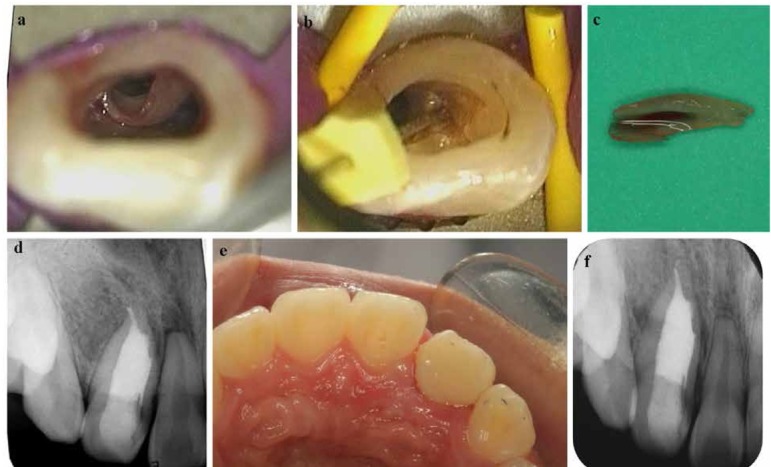
(a) The invaginated structure easily viewed under the DOM after troughing with the ultrasonic tip. (b) H-file used to engage the invaginated structure to remove it from the canal. (c) The removed invaginated structure. (d) IOPA taken after the obturation showing the completely filled canal. (e) Postoperative clinical view of the lingual surface after the restoration. (f) Two- year follow-up IOPA showing the healed lesion.

After local anesthesia, a rubber dam was positioned. With the help of DOM (OPMI PROergo; Carl Zeiss, Oberkochen, Germany), the endodontic access was started with a round diamond bur (ISO 801001016, Komet, Lemgo, Switzerland), followed with a long tapered diamond bur with round end (X-long tapered diamond, LA Axxess bur; SybronEndo, Orange, CA). The DI was troughed by using ET20 ultrasonic tip (P5, Satelec, France) carefully to separate the invaginated structure from the pulp canal space (Fig.2a). Once the DI was separated from the dentinal-enamel junction level, it was engaged with an H-file (Dentsply) (Fig 2b) and gently removed (Fig.2c). The gross granulation tissue after removal of DI in the canal was carefully debrided by ET18D ultrasonic tip (P5, Satele, France). The main root canal was prepared by Profile 35.06 (Dentsply/Maillefer). Subsequently, the root canal was irrigated with 5% sodium hypochlorite by using ET20 ultrasonic tip (P5, Satelec, France). Calcium hydroxide paste was placed into the canal for two weeks.

After two weeks, the patient was asymptomatic and the sinus tract was resolved. The working length 25.5mm was confirmed using apex locator (Root ZX; Morita, Tokyo, Japan). Final obturation of the root canals was performed with Schilder’s warm vertical compaction technique. The #35.06 GP master cone with AH-Plus sealer (Dentsply Maillefer) was placed in the canal. A heated plugger-Alpha II(Super Endo B&L, Korea) was used to remove portions of the coronal GP in successive increments and soften the remaining material in the canal. A plugger was inserted into the canal and the GP was compacted, forcing the plasticized material apically. The process was repeated until the apical portion was reached. The coronal canal space was back-filled using small preheated pieces of GP with the warm GP delivery system (Super Endo B&L, Korea). IOPA was taken after the obturation (Fig.2d). The final coronal restoration was accomplished using composite resin (Filtek Z 350, 3M ESPE, MN, USA) (Fig.2e). At the 2-year follow-up, the tooth was asymptomatic, and the periapical radiograph showed complete healing of periapical radiolucency of tooth #7 (Fig.2f).

Informed consent has been obtained from the patient and all individuals in this case report.

## 3 Discussion

Management of a tooth with DI is a great challenge considering the canal morphology and complex anatomy. The patient in this case report presented tooth #7 with DI (Oehlers type III) associated with pulp necrosis and chronic apical periodontitis. Disinfection of the DI was associated with the vigorous irrigation with 5% sodium hypochlorite for the diminution of endodontic biofilms in the root canal and promote antibacterial activity. Sodium hypochlorite at concentrations ranging from 0.5% to 5.25% and the intracanal medication calcium hydroxide are recommended by most of the authors [8, 16, 17]. There are many studies related to the removal of DI in maxillary lateral incisors [10, 11, 13]. Based on the literature review to date, there are no studies related to the removal of DI (type III) in maxillary lateral incisor with mature apex aided with CBCT analysis, DOI and ultrasonics, followed by successful endodontic treatment. Therefore, this illustrates the uniqueness and rareness of this present case report.

Successful endodontic treatment depends on the eradication of all the necrotic tissue along with bacterial irritants from the root canal system followed by obturation with a biocompatible material. It is known that teeth with DI exhibit a variation in canal morphologies, intracanal communications, inaccessible fins, and apical ramifications which are unreachable by many instruments and endodontic files [18]. The endodontist should be aware of the various techniques and materials and choose what is most convenient for the elimination of necrotic tissue from the root canal. A nonsurgical approach should always be considered before any surgical procedure [18]. In this case, the nonsurgical removal of DI was performed. Type III invaginations, as presented in this case report, are often difficult to treat as its complex root canal system prevents access to proper cleaning and shaping required for infection control. In the present case, the invagination invaded the root but also presented as a separate entity from the main root canal with a separate portal exit. Removal of the hard invaginated mass in the canal is difficult as well as challenging. Hence, the endodontist should choose an appropriate method to remove the hard deeply invaginated mass and necrotic tissue [9].

Minimal hand/rotary instrumentation is recommended in the DI due to the lack of need to shape the root canal system and also the presence of risk to weaken the tooth further [4]. Challenges that hinder successful biomechanical preparation and obturation are the inconsistent length and width of the canals with uncertain apical structure. Ultrasonic instrumentation which is desirable and recommended for the debridement of the infected tissues and debris to maximize their efficiency have been used in this case for the removable of the necrotic pulp tissue [19]. CBCT can be recommended as an effective diagnostic aid for DI because it provides an accurate representation of the external and internal dental anatomy of the tooth [20]. The unpredictable anomaly of DI is necessary to obtain a clear 3D picture of the complex invaginated structure. Additionally, a DOM helps in improved visualization and locating canals without loss of excess tooth structure. In order to prevent caries, direct infection, pulpal and periapical disease with the possible early loss of the tooth, the DI must be diagnosed early and the tooth must be restored. A radiograph can help detect DI prior to the eruption, and in the study by Thomas [20] it was recommended that DI teeth can prophylactically be restored at age 7 to 14.

## 4 Conclusion

Advancement in modern endodontic practice allows us to fulfil the technical and biological objective of endodontic treatment in a wide range of clinical cases through new treatment modalities. A clinician should have thorough knowledge regarding the morphologic, radiographic, clinical, and treatment aspects of a DI which may help as a preventive measure to preserve the vitality of the tooth. Thus, the present case illustrates that a tooth with DI and an associated periradicular lesion can be managed with nonsurgical removal of DI, with a satisfactory periradicular healing.

## Abbreviation

DI: Dens invaginatus
DOM: Dental operating microscope
CBCT: Cone-beam computed tomographic imaging
IOPA: Intraoral periapical radiograph
3D: Three-dimensional
2D: Two-dimensional
GP: Gutta-percha
